# In Silico Methods in Antibody Design

**DOI:** 10.3390/antib7030022

**Published:** 2018-06-29

**Authors:** Jun Zhao, Ruth Nussinov, Wen-Jin Wu, Buyong Ma

**Affiliations:** 1 Division of Biotechnology Review and Research I, Office of Biotechnology Products, Office of Pharmaceutical Quality, Center for Drug Evaluation and Research, US Food and Drug Administration, 10903 New Hampshire Avenue, Silver Spring, MD 20993, USA; Wen.Wu@fda.hhs.gov; 2 Interagency Oncology Task Force (IOTF) Fellowship: Oncology Product Research/Review Fellow, National Cancer Institute, Bethesda, MD 20892, USA; 3 Cancer and Inflammation Program, National Cancer Institute, Frederick, MD 21702, USA; 4 Basic Science Program, Leidos Biomedical Research, Inc. Cancer and Inflammation Program, National Cancer Institute, Frederick, MD 21702, USA; nussinor@mail.nih.gov (R.N.); buyong.ma@nih.gov (B.M.); 5 Sackler Inst. of Molecular Medicine, Department of Human Genetics and Molecular Medicine, Sackler School of Medicine, Tel Aviv University, Tel Aviv 69978, Israel

**Keywords:** antibody design, epitope prediction, antibody-antigen recognition, affinity maturation, immunogenicity, vaccine design, antibody stability, allosteric effect

## Abstract

Antibody therapies with high efficiency and low toxicity are becoming one of the major approaches in antibody therapeutics. Based on high-throughput sequencing and increasing experimental structures of antibodies/antibody-antigen complexes, computational approaches can predict antibody/antigen structures, engineering the function of antibodies and design antibody-antigen complexes with improved properties. This review summarizes recent progress in the field of in silico design of antibodies, including antibody structure modeling, antibody-antigen complex prediction, antibody stability evaluation, and allosteric effects in antibodies and functions. We listed the cases in which these methods have helped experimental studies to improve the affinities and physicochemical properties of antibodies. We emphasized how the molecular dynamics unveiled the allosteric effects during antibody-antigen recognition and antibody-effector recognition.

## 1. Introduction

**Figure 1:**
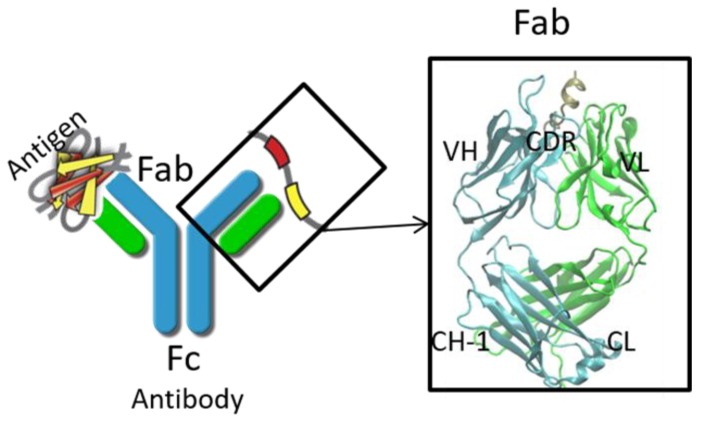
Schematic representation of an antibody with Fab region and Fc region. Light chain and heavy chain of the antibody are colored in cyan and green. CDR: complementarity determining region; VH: heavy chain variable domain; VL: light chain variable domain; CL: light chain constant domain; CH-1: heavy chain constant domain-1.

Nowadays, monoclonal immunoglobulin gamma (IgG) antibodies have become a major framework in cancer therapy and therapy for many other critical diseases. IgG molecules bind to their cognate antigens and the immune complexes subsequently interact either with type I or type II Fc receptors on effector cells and on B cells, modulating both humoral immune processes and innate immune processes [1]. Structurally, IgG contains four polypeptide chains, including two light chains (LC) and two heavy chains (HC). The four chains fold into three domains (Figure 1), that is, two Fab domains, which bind antigen and one Fc domain, which bind Fc receptors for effector function [2]. The Fab domains contain variable domain and constant domain. The variable domains, especially complementarity determining regions (CDRs), are mainly responsible for specificity and affinity [3], while the constant domains modulate the isotype/effector functions [4]. The Fc domain contains CH2 and CH3 domains. The CH2 domain mainly interacts with Fc receptors (FcRs), which are on the cell surface and play pivotal roles in humoral and cellular protection. Other regions, including the hinge region and glycan, also affect antibody activities, (e.g., binding [5], pharmacokinetics [6], and effector functions [7]).

Interestingly, numerous evidences have shown that the antibody constant domain also plays a role in antibody antigen recognition [8]. Antibodies with identical V regions differing in isotype or subclass manifest either differences in antigen binding [9,10] or altered specificity [11,12]. Engineering the above portion of the antibody will optimize the properties of the antibody with the desired efficacy. The available crystal structures of antibodies, antibody-antigen complexes, and Fc/Fc receptor complexes are increasing. Meanwhile, computational resources are increasing and algorithms, and molecular mechanics force fields are becoming more accurate to model molecular behaviors, especially local rearrangement. Thus, the in silico molecular modeling techniques are becoming popular to engineer antibodies [13], (e.g., Fc-based antibody domains and fragments [14], disulfide bonds [15], and T-cell receptor(TCR) mimic antibodies [16] with desired properties, such as viscosity and phase separation properties [17]). In this review, we focused on the reports using molecular modeling in antibody design and the study of antibody behaviors, especially the allosteric effects related to antibody/antigen recognition.

## 2. Structure Prediction of Variable Domain and Complementarity-Determining Regions

Complementarity-determining regions (CDRs) are part of the variable chains in IgGs, and a set of CDRs constitutes a paratope. Chothia and Lesk define the “hypervariable loops” based on the relationship between amino acid sequences and three-dimensional structures around the antigen binding region [18,19]. They found that five out of six hypervariable regions, (i.e., light chain CDR loop 1–3 (L1, L2, L3) and heavy chain CDR loop 1–2 (H1 and H2)) typically adopt a limited number of discrete backbone conformations, called “canonical structures”. They further identified several residues that are responsible for the main-chain conformations of the hypervariable loops. To build the reliable antibody variable fragment (Fv) structures, people have extensively studied the conformational library of canonical structures [20,21,22,23,24]. Within the same type of canonical structure, the average backbone root-mean-square deviation (RMSD) between a target loop and a template loop is approximately 0.7 Å. It should be noted that the above canonical structures work well in human and murine antibodies but not in other organisms, such as the camelid heavy chain antibodies, which only have varied domain of heavy chain [25], and bovine antibodies, which have ultra-long VH CDR3 regions [26]. Based on the canonical structures, the structures of antibodies can be modeled by careful selection of at least eight templates, including two for the light and heavy chain frameworks selected by sequence similarity, five templates for non-H3 loops selected using the canonical structures and one for the H3 loop partially based on the ab initio methods [27,28] (e.g., PLOP [29], Modeller [30], Loopy [31], and the loop modeling in Rosetta [32]). Different from the canonical structure approach, ab initio methods depend on conformation predictions using physicochemical principles rather than using structural templates. Different from the canonical structure approach, ab initio methods depend on physicochemical principles rather than the template. Due to the simplification of the energy functions and the limited computational resources, the ab initio methods have limitation in accuracy.

Several widely used antibody structure prediction methods are developed from the industry, (e.g., Chemical Computer Group (CCG), Schrödinger Inc. (New York, NY, USA) [33] and Accelrys Inc. (San Diego, CA, USA)) or academia, (e.g., PIGS server [27]). These methods combined different degrees of automation, template selection criteria, types of energy functions, and sampling algorithms. To evaluate the reliability of the predictions compared to the experimentally determined structure of the antibody, the antibody modeling assessment (AMA) initiated an evaluation platform to compare the antibody structures from experiments and modeling [34,35]. AMA used unpublished, high-resolution Fab crystal structures as a benchmark to compare models generated by the different methods. After the evaluation, AMA-II concluded that although there has been great progress in the prediction of antibody structure, high-quality experimental structures are still the most important for modeling antibody structures with high accuracy. Moreover, a combination of homology modeling with knowledge-based and energy-based methods can generate more reliable H3 loops [36]. For example, RosettaAntibody [37,38] combined homology and ab initio modeling to build a preliminary homology model by selecting different templates for the frameworks and non-H3 CDRs, modeling the H3 loop and optimizing the heavy chain variable domain (VH)/light chain variable domain (VL) interface using ab initio methods.

## 3. Antibody-Antigen Binding Prediction, Epitope Mapping, and Affinity Maturation

Using the three-dimensional structures of the antibody-antigen complexes, it is possible to enhance the antibody-antigen binding affinities by in silico mutations on antibody residues. In the best situation, when the antibody-antigen complex structures are available, it is relatively straight-forward to perform affinity maturation in silico. Firstly, as an initial step, the protein backbone was treated as rigid, and the conformation of the side chain was determined by discrete side-chain rotamer search. Secondly, the lowest-energy of the structures was further re-evaluated by using more accurate, but computationally more expense models, (e.g., Poisson–Boltzmann (PB) or Generalized Born (GB) continuum electrostatics, unbound-state side-chain conformation search, and minimization). Based on the crystal complex structure between 11K2 and MCP-1 [39], all residues of the CDRs of 11K2 were systematically mutated to 19 other natural amino acids computationally. The interaction energy between the antigen and the antibody was then evaluated and 12 mutations showed improved in silico binding energy. The binding affinity of these mutants were further evaluated by surface plasmon resonance (SPR). Among the 12 mutants, five showed improved binding affinity and one showed a 4.6-fold improvement. A 10 times increase in affinity was achieved by Lippow et al. by redesigning an antilysozyme antibody [40]. Interestingly, Lippow et al. showed that computed electrostatics alone is better than computed total free energy to improve binding [40] in that specific case. Their results suggested that using only electrostatics interaction could be a less expensive but more accurate indicator to predict the binding affinity between antibody and antigen.

One of the significant challenges in modeling antibody antigen complexes is docking the antibody onto its epitope on the surface of the antigen. As epitopes and paratopes are typically flat, the shape complementarity between antibody and antigen is not a good determinant of correct antibody placement, which limits the application of general protein-protein docking procedures. SnugDock [41], which is based on the RosettaDock algorithm [42], applied alternating rounds of low-resolution rigid body perturbations and high-resolution side-chain and backbone minimization to generate a model of the antibody-antigen complex. The protocol relies on random perturbation of the complex and creates large numbers (∼10^5^) of models to capture a global energy minimum. Encouragingly, the antibody-antigen complexes showed a strong energy funnel, with low energy structures corresponding to a low RMSD to the native structure, indicating that this method can recover the native conformation. Zhao et al. used RosettaDock to search the possible complexes between Aβ fibrils/oligomers and a therapeutic antibody [43], crenuzumab, which is designed to reduce the Aβ species in blood. They selected five Aβ oligomer-crenuzumab complexes and refined the docked conformation using molecular dynamics (MD) simulations. They found that two out of five remain stable, which explain the experimental observation of the antibody’s recognition of amyloid. The results illustrated the usefulness of further refinements of docking results by MD simulation.

An alternative computational approach to physical modeling is the knowledge-based residue pair preference on epitope–paratope interfaces. With the increasing crystal structures of antibody-antigen complexes in the Protein Data Bank (PDB), a statistical amino acid interaction preference matrix can be used to predict the antibody-antigen recognition. Wang et al. studied the physicochemical properties of regions on and far from the antibody-antigen interfaces, such as net charge, overall antibody charge distributions, and their role in antigen interactions. They found that amino acid preference in antibody-protein antigen recognition is entropy driven. The interface residues with low side-chain entropy are selected to compensate for the high backbone entropy when interacting with protein antigens Positively charged and polar antigen residues and bridging water molecules have a higher possibility of being selected on the antibody-protein antigen interface. Tyr, Ser, and Asp but few Lys are selected on the antibody-antigen interfaces [44]. K. Tharakaraman et al. generated a similar matrix using the available crystal structure. They applied the matrix to guide the antibody-antigen docking of antibody 4E11, which has no crystal structure [45]. They designed affinity-enhancing mutations with a 450-fold improvement, leading to a potent cross-reactive neutralizing antibody to target dengue virus [45].

Although there are many successful cases for protein-protein affinity design, there are still challenges as the antibody-antigen recognition is not simply interactions between proteins; the solvent effect is also crucial. For instance, interfacial trapped water molecules, polar and charged side chains, and the balance between protein-solvent and protein-protein interactions from the unbound to bound state need to be considered during the modeling.

## 4. Antibody Aggregation, Stability, and Immunogenicity

In high concentrations of formulations for therapy or storage [46], antibodies have been known to aggregate, and the aggregation of therapeutic proteins can lead to immunogenicity [47,48,49]. Numerous experimental studies have been performed to investigate antibody stability and resistance to aggregation [50,51,52,53], especially in single-chain Fv fragments. Molecular modeling is a useful tool in addition to experimental techniques to predict aggregate-prone regions (APRs) [46], and to design aggregation-resistant antibodies by introducing mutations in those regions. The sequence composition and several structural properties, such as hydrophobicity, charge, and secondary structure propensity, were used to predict the APRs [54,55,56,57]. The experimental datasets were also able to predict the aggregation rate upon different mutations [58,59]. Wang et al. investigated the APRs on the CDR region, and they found that the APRs most frequently appeared in CDR-H2 and less frequently in CDR-H3 [60]. Moreover, they showed that aromatic residues (e.g., Tyr and Trp) are favored both on CDR and APRs, indicating that co-incidence of APRs with CDR sites can potentially cause the loss of function upon aggregation. Trout et al. quantified the exposure of hydrophobic residues averaged over snapshots from MD simulations, and they used this value as a novel indicator (i.e., spatial aggregation propensity (SAP) to predict APRs) [61,62]. They identified 14 aggregation-prone motifs in constant regions of human IgG molecules, which are not conserved among the other antibody classes [63].

**Figure 2:**
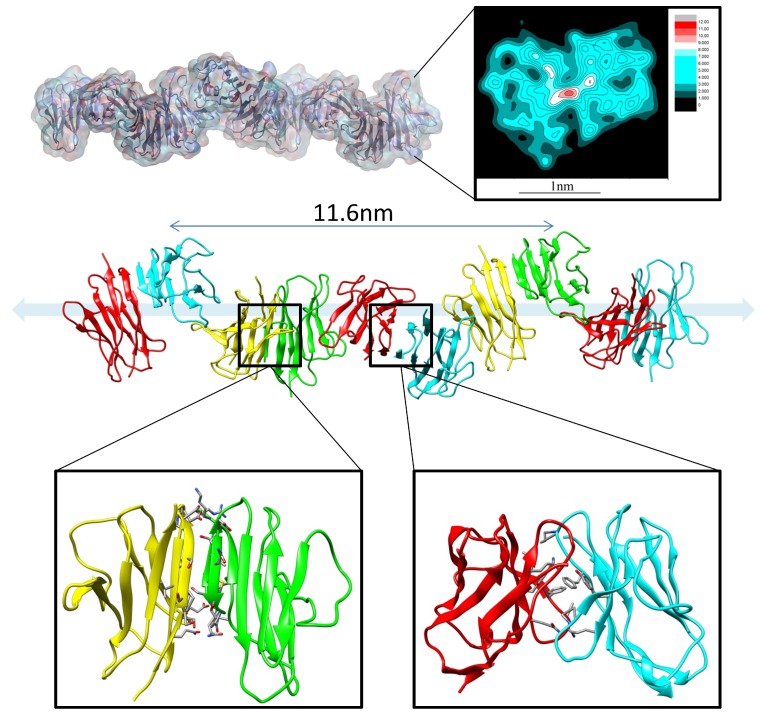
The canonical dimer interface is essential for amyloidosis (AL) fibril formation. The fibril structure of AL with the partially misfolded protomer and native protomer.

MD simulations have been used to study the mechanisms of antibody aggregation and amyloidosis [64,65]. In the physiological condition, amyloid formation and deposition of immunoglobulin light-chain proteins in systemic amyloidosis (AL) cause major organ failures [66,67,68]. While the κ light-chain is dominant (λ/κ = 1:2) in healthy individuals, λ is overrepresented (λ/κ = 3:1) in AL patients [69]. To understand the structural basis of the amyloid formation and the sequence preference, Zhao et al. examined the correlation between the sequence and structural stability of dimeric variable domains of immunoglobulin light chains using molecular dynamics simulations of 24 representative dimer interfaces, followed by energy evaluation of conformational ensembles for 20 AL patients’ light-chain sequences. They identified a stable interface with displaced N-terminal residues. This interface provides the structural basis for AL protein fibrils formation (Figure 2). Proline isomerization may cause the N-terminus to adopt amyloid-prone conformations. They found that λ light chains prefer misfolded dimer conformation, while κ chain structures are stabilized by a natively folded dimer. According to the available crystal structures, the structural repertoire of λ chains is different and more diverse than that κ of the chains [22]. This suggested that λ light-chain protomers have larger conformational diversity than κ chain protomers and thus are easier to be unfolded. The unfolded λ light-chain protomer further formed aggregates, while the intact κ chain protomer can be protected by the natively folded dimer.

## 5. Allosteric Effects in Antibodies

**Figure 3:**
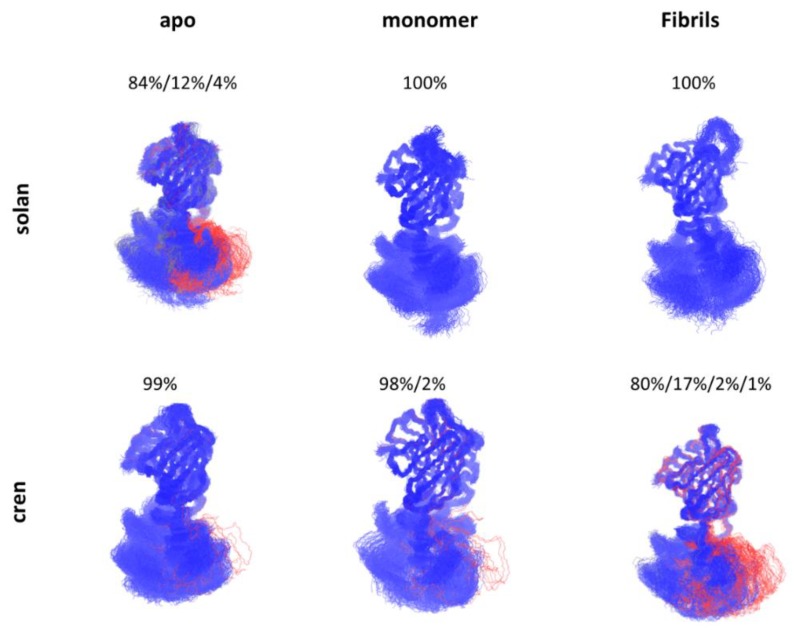
Allosteric effect in antibody-antigen recognition. The constant domain of the Fab region of solanuzbmab and crenezumab response to the Aβ binding differently with conformational shifts.

The process of antibody-antigen recognition is complicated and associated with conformational transitions of the antibody by the inherent flexibility [70,71,72]. Recent studies showed that there are allosteric effects during the recognition process of antibody-antigen recognition [73,74]. Interestingly, the constant domain also plays an essential role in antigen recognition [8,75,76,77,78,79]. Based on over 100 crystal structures of antibody Fab domains in either unbound or bound form, a common effect was discovered, that is, distant CH1-1 loops undergo significant fluctuation upon antigen binding [80]. The removal of the intermolecular disulfide bond between light chain and heavy chain in a Fab-recognizing prion peptide showed binding energy enhancement upon the molecular dynamics simulation [81]. Zhao et al.’s work on solanuzumab and crenezumab showed that antibodies with identical variable domain, but different constant domain have significantly different affinities when binding to Aβ species [43]. Interestingly, they found that in the apo form, the constant domain of solanuzumab is more flexible than crenezumab while more rigid than crenezumab when bound to Aβ fibrils, and this flexibility change might correspond to the binding affinity difference between crenezumab and solanuzumab (Figure 3). They further proposed that this flexibility change is potentially due to the entropy redistribution after the antibody-antigen recognition.

## 6. Modulation of the Effector Functions

Besides antigen recognition, the effector functions, such as antibody-dependent cellular cytotoxicity, complement-dependent cytotoxicity, and antibody-dependent cell-mediated phagocytosis, can also be engineered based on the structures between fragment crystallizable region (Fc) and receptors and the computational techniques in a high-throughput way. The engineerable parts are the hinge region, CH2 domain, N-glycans and N-glycan-attached residues. All’acqua et al. introduced various modifications into the hinge region of mAb 12G3H11 to modulate the hinge’s length, flexibility, and/or biochemical properties. They found that the upper and middle hinge are important, and mutations introduced to these regions can modulate the FcγRIIIa or C1q binding [5]. Lazar et al. applied computational optimization of the Fc region [82] using Protein Design Automation (PDA) [83] technology and Sequence Prediction Algorithm (SPA) algorithm [84]. They created various mutations to improve the affinity up to 169-fold with a FcγRIIIa:IIb ratio about nine-fold. Engineering on the N-glycan can also modulate the effector functions. The glycans at Asn-297 (N-glycan) are important to maintain the quaternary structure and the stability of the Fc [85], as well as the Fc-Fc receptor recognition [86,87,88,89]. The deglycosylation of IgG1 resulted in a 40-fold loss in FcγRI binding [86]. The composition of N-glycans can modulate the binding affinity of IgG1 Fc to Fc γ receptors [90,91,92,93]. Lee et al. applied molecular dynamics to the Fc region of an antibody, and they found that the C’E loop and the CH2-CH3 orientation are dynamic and changes in N-glycan composition shift their conformational ensembles and optimize the interface with the Fc receptor for efficient binding [94]. The noncovalent interactions of multiple amino acid residues from the Fc domain with the N-glycan are necessary for optimal recognition of the protein-binding site by FcγRI [95]. Moreover, single amino acid mutations at these residues of contact from the Fc domain have considerable effects on glycan processing [95,96,97]. X-ray crystallography and NMR data indicated that the two arms of N-glycan showed either the bound state (attached to the Fc fragment) [98] or the free state (detached from the Fc fragment) [99]. Several studies showed that mutations on the residues, which bind to the N-glycan, can shift the free/bound states and then modulate the effector function [100].

## 7. In Silico Vaccine Design

In silico methods are also widely used in vaccine design. Generally, structure-based in silico vaccine design includes epitope identification, immunogen design by epitope grafting, and antibody/antigen structure prediction. The epitopes include linear (both T-cell and B-cell) and conformational epitopes (mainly B-cell). The bioinformatics tools are well-established to predict the linear epitope [101,102,103,104]. For the conformational (discontinuous) epitopes, the three-dimensional (3D) structure of proteins is often required (e.g., envelope glycoproteins [105,106,107], epitope peptides [108], and the native viral spike [109,110]). These methods use the 3D structure to determine the physico-chemical properties, such as the surface accessibility, the propensity scores of residues in spatial proximity, and the contact numbers of residues, to obtain the final score for epitope evaluation [111,112,113]. In case of no available crystal structure, in silico protein structure prediction is useful. These methods, including template-based and free modeling, are similar to the antibody structure prediction and antibody-antigen complex structure prediction in the previous section, but with a focus on the antigen part. Ideally, among the epitope candidates, the selected epitopes in a vaccine should be conserved across different stages of the pathogen and its variants with the consideration of desired immune response. The epitope can be grafted onto a heterologous protein scaffold for immunogen design [114]. There are three criteria for selecting suitable scaffolds for epitope grafting [115]. Firstly, smaller epitopes are preferred to minimize the unwanted immunogenicity. Secondly, flexibility of the epitope can be tuned to improve the wanted immunogenicity. Thirdly, the neighboring residues are important for the specificity. Besides the epitope, the non-epitope regions should also be resurfaced to enhance the immunogen properties, (e.g., solubility and stability [116,117]). There are several successful cases of in silico design of vaccines. For example, Correia et al. started the vaccine discovery by computational protein design [118]. They generated small, thermally and conformationally stable protein scaffolds by the in silico methods. Further experiments showed that these protein scaffolds accurately mimic the viral epitope structure and induce potent neutralizing antibodies.

## 8. Conclusions

High-throughput technology combined with computational technologies have dramatically advanced the development of biological therapeutics [119,120,121]. In the field of antibody therapeutics, there are various antibody data resources in terms of their contents and features including PDB, DIGIT, IEDB, and IMGT. To engineering antibodies, the 3D structures are crucial to understand the antibody-antigen recognition mechanism, to evaluate the stability and immunogenicity of the antibody, and to predict the function/efficacy change upon modification. However, the available experimental techniques, such as crystallography and NMR, can only solve small portion of the protein structure space. Computational methods can expand this space by selecting reasonable templates, predicting epitope, and modeling the CDR region with acceptable deviation. However, the combination of these computational capabilities might result in accumulated errors, especially for the antibody-antigen complex structure prediction [122]. Recent critical assessment of protein interactions(CAPRI) reported that six out of 20 test complexes cannot obtain acceptable models even with ~1000 candidate models per complex [123]. Thus, in the case of protein without available structure or template, it is still challenging to perform the whole design process in silico.

Improvements in conformational sampling methods and development of scoring functions used to estimate free energies are also much-needed. Sampling the conformation of flexible CDR loops, especially CDR-H3, is particularly important in antibody design, because large conformational changes can occur when antigen binding. Moreover, the evaluation of antibody allosteric effect requires extensive sampling about the motion and dynamic of antibody constant domains. Free energy perturbation (FEP) methodology can be used to estimate relative antigen binding affinity differences to the antibody variants [124], but the set-up of these calculations is tedious and the simulations are time-consuming (about 1–2 days per structure), which limits its applicability to only small number of structures. Empirical scoring functions [125,126,127,128,129] serve as alternative methods to free energy simulation methods, offering a quicker way to estimate binding affinities where high computational throughput is needed (Ala scanning or affinity maturation) [130]. The lower accuracy and applicability of the empirical scoring functions needs to be considered in practical applications.

Allosteric effects and conformational change in antibody-antigen recognition and antibody effector functions are emerging and challenging to evaluate using experimental techniques [131,132,133]. Molecular dynamics simulations can be used to study the allosteric effects; however, they require infeasible sampling time using regular sampling techniques. The emerging Markov State Models [134] can be used to create conformational free energy profiles with multiple shorter simulations to better evaluate the allosteric effects [135]. To summarize, the combination of in silico technologies, expending databases, and greater availability of structures of antibody-antigen complexes will have a real impact in aiding antibody drug discovery.

## References

[1] Pincetic A., Bournazos S., DiLillo D.J., Maamary J., Wang T.T., Dahan R., Fiebiger B.M., Ravetch J.V. (2014). Type I and type II Fc receptors regulate innate and adaptive immunity. Nat. Immunol..

[2] Schroeder H.W., Cavacini L. (2010). Structure and function of immunoglobulins. J. Allergy Clin. Immunol..

[3] Mian I.S., Bradwell A.R., Olson A.J. (1991). Structure, function and properties of antibody binding sites. J. Mol. Biol..

[4] Torres M., Casadevall A. (2008). The immunoglobulin constant region contributes to affinity and specificity. Trends Immunol..

[5] Dall’Acqua W.F., Cook K.E., Damschroder M.M., Woods R.M., Wu H. (2006). Modulation of the effector functions of a human IgG1 through engineering of its hinge region. J. Immunol..

[6] Higel F., Seidl A., Sorgel F., Friess W. (2016). *N*-glycosylation heterogeneity and the influence on structure, function and pharmacokinetics of monoclonal antibodies and Fc fusion proteins. Eur. J. Pharm. Biopharm..

[7] Flynn G.C., Chen X., Liu Y.D., Shah B., Zhang Z. (2010). Naturally occurring glycan forms of human immunoglobulins G1 and G2. Mol. Immunol..

[8] Janda A., Bowen A., Greenspan N.S., Casadevall A. (2016). Ig constant region effects on variable region structure and function. Front. Microbiol..

[9] Tomaras G.D., Ferrari G., Shen X., Alam S.M., Liao H.X., Pollara J., Bonsignori M., Moody M.A., Fong Y., Chen X. (2013). Vaccine-induced plasma IgA specific for the C1 region of the HIV-1 envelope blocks binding and effector function of IgG. Proc. Natl. Acad. Sci. USA.

[10] Cooper L.J., Shikhman A.R., Glass D.D., Kangisser D., Cunningham M.W., Greenspan N.S. (1993). Role of heavy chain constant domains in antibody-antigen interaction. Apparent specificity differences among streptococcal IgG antibodies expressing identical variable domains. J. Immunol..

[11] Kato K., Matsunaga C., Odaka A., Yamato S., Takaha W., Shimada I., Arata Y. (1991). Carbon-13 NMR study of switch variant anti-dansyl antibodies: Antigen binding and domain-domain interactions. Biochemistry.

[12] Torres M., May R., Scharff M.D., Casadevall A. (2005). Variable-region-identical antibodies differing in isotype demonstrate differences in fine specificity and idiotype. J. Immunol..

[13] Gilliland G.L., Luo J., Vafa O., Almagro J.C. (2012). Leveraging SBDD in protein therapeutic development: Antibody engineering. Methods Mol. Biol..

[14] Ying T., Gong R., Ju T.W., Prabakaran P., Dimitrov D.S. (2014). Engineered fc based antibody domains and fragments as novel scaffolds. Biochim. Biophys. Acta.

[15] Hagihara Y., Saerens D. (2014). Engineering disulfide bonds within an antibody. Biochim. Biophys. Acta.

[16] Chang A.Y., Gejman R.S., Brea E.J., Oh C.Y., Mathias M.D., Pankov D., Casey E., Dao T., Scheinberg D.A. (2016). Opportunities and challenges for TCR mimic antibodies in cancer therapy. Expert Opin. Biol. Ther..

[17] Chow C.K., Allan B.W., Chai Q., Atwell S., Lu J. (2016). Therapeutic antibody engineering to improve viscosity and phase separation guided by crystal structure. Mol. Pharm..

[18] Chothia C., Lesk A.M. (1987). Canonical structures for the hypervariable regions of immunoglobulins. J. Mol. Biol..

[19] Chothia C., Lesk A.M., Tramontano A., Levitt M., Smith-Gill S.J., Air G., Sheriff S., Padlan E.A., Davies D., Tulip W.R. (1989). Conformations of immunoglobulin hypervariable regions. Nature.

[20] North B., Lehmann A., Dunbrack R.L. (2011). A new clustering of antibody CDR loop conformations. J. Mol. Biol..

[21] Al-Lazikani B., Lesk A.M., Chothia C. (1997). Standard conformations for the canonical structures of immunoglobulins. J. Mol. Biol..

[22] Chailyan A., Marcatili P., Cirillo D., Tramontano A. (2011). Structural repertoire of immunoglobulin lambda light chains. Proteins.

[23] Kuroda D., Shirai H., Kobori M., Nakamura H. (2009). Systematic classification of CDR-L3 in antibodies: Implications of the light chain subtypes and the VL-VH interface. Proteins.

[24] Martin A.C., Thornton J.M. (1996). Structural families in loops of homologous proteins: Automatic classification, modelling and application to antibodies. J. Mol. Biol..

[25] Sircar A., Sanni K.A., Shi J., Gray J.J. (2011). Analysis and modeling of the variable region of camelid single-domain antibodies. J. Immunol..

[26] Wang F., Ekiert D.C., Ahmad I., Yu W., Zhang Y., Bazirgan O., Torkamani A., Raudsepp T., Mwangi W., Criscitiello M.F. (2013). Reshaping antibody diversity. Cell.

[27] Marcatili P., Rosi A., Tramontano A. (2008). Pigs: Automatic prediction of antibody structures. Bioinformatics.

[28] Whitelegg N.R., Rees A.R. (2000). WAM: An improved algorithm for modelling antibodies on the web. Protein Eng..

[29] Jacobson M.P., Pincus D.L., Rapp C.S., Day T.J., Honig B., Shaw D.E., Friesner R.A. (2004). A hierarchical approach to all-atom protein loop prediction. Proteins.

[30] Fiser A., Do R.K., Sali A. (2000). Modeling of loops in protein structures. Protein Sci..

[31] Xiang Z., Soto C.S., Honig B. (2002). Evaluating conformational free energies: The colony energy and its application to the problem of loop prediction. Proc. Natl. Acad. Sci. USA.

[32] Stein A., Kortemme T. (2013). Improvements to robotics-inspired conformational sampling in rosetta. PLoS ONE.

[33] Zhu K., Day T., Warshaviak D., Murrett C., Friesner R., Pearlman D. (2014). Antibody structure determination using a combination of homology modeling, energy-based refinement, and loop prediction. Proteins.

[34] Almagro J.C., Teplyakov A., Luo J., Sweet R.W., Kodangattil S., Hernandez-Guzman F., Gilliland G.L. (2014). Second antibody modeling assessment (AMA-II). Proteins.

[35] Almagro J.C., Beavers M.P., Hernandez-Guzman F., Maier J., Shaulsky J., Butenhof K., Labute P., Thorsteinson N., Kelly K., Teplyakov A. (2011). Antibody modeling assessment. Proteins.

[36] Martin A.C., Cheetham J.C., Rees A.R. (1989). Modeling antibody hypervariable loops: A combined algorithm. Proc. Natl. Acad. Sci. USA.

[37] Sircar A., Kim E.T., Gray J.J. (2009). Rosettaantibody: Antibody variable region homology modeling server. Nucleic Acids Res..

[38] Weitzner B.D., Kuroda D., Marze N., Xu J., Gray J.J. (2014). Blind prediction performance of rosettaantibody 3.0: Grafting, relaxation, kinematic loop modeling, and full CDR optimization. Proteins.

[39] Kiyoshi M., Caaveiro J.M., Miura E., Nagatoishi S., Nakakido M., Soga S., Shirai H., Kawabata S., Tsumoto K. (2014). Affinity improvement of a therapeutic antibody by structure-based computational design: Generation of electrostatic interactions in the transition state stabilizes the antibody-antigen complex. PLoS ONE.

[40] Lippow S.M., Wittrup K.D., Tidor B. (2007). Computational design of antibody-affinity improvement beyond in vivo maturation. Nat. Biotechnol..

[41] Sircar A., Gray J.J. (2010). Snugdock: Paratope structural optimization during antibody-antigen docking compensates for errors in antibody homology models. PLoS Comput. Biol..

[42] Gray J.J., Moughon S., Wang C., Schueler-Furman O., Kuhlman B., Rohl C.A., Baker D. (2003). Protein-protein docking with simultaneous optimization of rigid-body displacement and side-chain conformations. J. Mol. Biol..

[43] Zhao J., Nussinov R., Ma B. (2017). Mechanisms of recognition of amyloid-β (Aβ) monomer, oligomer, and fibril by homologous antibodies. J. Biol. Chem..

[44] Wang M., Zhu D., Zhu J., Nussinov R., Ma B. (2017). Local and global anatomy of antibody-protein antigen recognition. J. Mol. Recognit..

[45] Tharakaraman K., Robinson L.N., Hatas A., Chen Y.L., Siyue L., Raguram S., Sasisekharan V., Wogan G.N., Sasisekharan R. (2013). Redesign of a cross-reactive antibody to dengue virus with broad-spectrum activity and increased in vivo potency. Proc. Natl. Acad. Sci. USA.

[46] Ventura S., Zurdo J., Narayanan S., Parreno M., Mangues R., Reif B., Chiti F., Giannoni E., Dobson C.M., Aviles F.X. (2004). Short amino acid stretches can mediate amyloid formation in globular proteins: The Src homology 3 (SH3) case. Proc. Natl. Acad. Sci. USA.

[47] Rosenberg A.S. (2006). Effects of protein aggregates: An immunologic perspective. AAPS J..

[48] Kumar S., Singh S.K., Wang X., Rup B., Gill D. (2011). Coupling of aggregation and immunogenicity in biotherapeutics: T- and b-cell immune epitopes may contain aggregation-prone regions. Pharm. Res..

[49] Worn A., Pluckthun A. (2001). Stability engineering of antibody single-chain Fv fragments. J. Mol. Biol..

[50] Bird R.E., Hardman K.D., Jacobson J.W., Johnson S., Kaufman B.M., Lee S.M., Lee T., Pope S.H., Riordan G.S., Whitlow M. (1988). Single-chain antigen-binding proteins. Science.

[51] Glockshuber R., Malia M., Pfitzinger I., Pluckthun A. (1990). A comparison of strategies to stabilize immunoglobulin Fv-fragments. Biochemistry.

[52] Worn A., Pluckthun A. (1998). Mutual stabilization of VL and VH in single-chain antibody fragments, investigated with mutants engineered for stability. Biochemistry.

[53] Worn A., Pluckthun A. (1999). Different equilibrium stability behavior of ScFv fragments: Identification, classification, and improvement by protein engineering. Biochemistry.

[54] Caflisch A. (2006). Computational models for the prediction of polypeptide aggregation propensity. Curr. Opin. Chem. Biol..

[55] Conchillo-Sole O., de Groot N.S., Aviles F.X., Vendrell J., Daura X., Ventura S. (2007). Aggrescan: A server for the prediction and evaluation of “hot spots” of aggregation in polypeptides. BMC Bioinform..

[56] Trovato A., Seno F., Tosatto S.C. (2007). The pasta server for protein aggregation prediction. Protein Eng. Des. Sel..

[57] Garbuzynskiy S.O., Lobanov M.Y., Galzitskaya O.V. (2010). Foldamyloid: A method of prediction of amyloidogenic regions from protein sequence. Bioinformatics.

[58] DuBay K.F., Pawar A.P., Chiti F., Zurdo J., Dobson C.M., Vendruscolo M. (2004). Prediction of the absolute aggregation rates of amyloidogenic polypeptide chains. J. Mol. Biol..

[59] Chiti F., Stefani M., Taddei N., Ramponi G., Dobson C.M. (2003). Rationalization of the effects of mutations on peptide and protein aggregation rates. Nature.

[60] Wang X., Singh S.K., Kumar S. (2010). Potential aggregation-prone regions in complementarity-determining regions of antibodies and their contribution towards antigen recognition: A computational analysis. Pharm. Res..

[61] Chennamsetty N., Voynov V., Kayser V., Helk B., Trout B.L. (2009). Design of therapeutic proteins with enhanced stability. Proc. Natl. Acad. Sci. USA.

[62] Voynov V., Chennamsetty N., Kayser V., Helk B., Trout B.L. (2009). Predictive tools for stabilization of therapeutic proteins. MAbs.

[63] Chennamsetty N., Helk B., Voynov V., Kayser V., Trout B.L. (2009). Aggregation-prone motifs in human immunoglobulin G. J. Mol. Biol..

[64] Ma B., Nussinov R. (2006). Simulations as analytical tools to understand protein aggregation and predict amyloid conformation. Curr. Opin. Chem. Biol..

[65] Sharma S., Ding F., Dokholyan N.V. (2008). Probing protein aggregation using discrete molecular dynamics. Front. Biosci..

[66] Wall J., Schell M., Murphy C., Hrncic R., Stevens F.J., Solomon A. (1999). Thermodynamic instability of human lambda 6 light chains: Correlation with fibrillogenicity. Biochemistry.

[67] Stevens P.W., Raffen R., Hanson D.K., Deng Y.L., Berrios-Hammond M., Westholm F.A., Murphy C., Eulitz M., Wetzel R., Solomon A. (1995). Recombinant immunoglobulin variable domains generated from synthetic genes provide a system for in vitro characterization of light-chain amyloid proteins. Protein Sci..

[68] Kim Y., Wall J.S., Meyer J., Murphy C., Randolph T.W., Manning M.C., Solomon A., Carpenter J.F. (2000). Thermodynamic modulation of light chain amyloid fibril formation. J. Biol. Chem..

[69] Kyle R.A., Gertz M.A. (1995). Primary systemic amyloidosis: Clinical and laboratory features in 474 cases. Semin. Hematol..

[70] Keskin O. (2007). Binding induced conformational changes of proteins correlate with their intrinsic fluctuations: A case study of antibodies. BMC Struct. Biol..

[71] Thielges M.C., Zimmermann J., Yu W., Oda M., Romesberg F.E. (2008). Exploring the energy landscape of antibody−antigen complexes: Protein dynamics, flexibility, and molecular recognition. Biochemistry.

[72] Li T., Tracka M.B., Uddin S., Casas-Finet J., Jacobs D.J., Livesay D.R. (2014). Redistribution of flexibility in stabilizing antibody fragment mutants follows le chatelier’s principle. PLoS ONE.

[73] Sela-Culang I., Kunik V., Ofran Y. (2015). The structural basis of antibody-antigen recognition. Immune Syst. Model. Anal..

[74] Jay J.W., Bray B., Qi Y., Igbinigie E., Wu H., Li J., Ren G. (2018). IgG antibody 3D structures and dynamics. Antibodies.

[75] Adachi M., Kurihara Y., Nojima H., Takeda-Shitaka M., Kamiya K., Umeyama H. (2003). Interaction between the antigen and antibody is controlled by the constant domains: Normal mode dynamics of the hel–hyhel-10 complex. Protein Sci..

[76] Pritsch O., Hudry-Clergeon G., Buckle M., Pétillot Y., Bouvet J.P., Gagnon J., Dighiero G. (1996). Can immunoglobulin C (H) 1 constant region domain modulate antigen binding affinity of antibodies?. J. Clin. Investig..

[77] Dam T.K., Torres M., Brewer C.F., Casadevall A. (2008). Isothermal titration calorimetry reveals differential binding thermodynamics of variable region-identical antibodies differing in constant region for a univalent ligand. J. Biol. Chem..

[78] Tudor D., Yu H., Maupetit J., Drillet A.-S., Bouceba T., Schwartz-Cornil I., Lopalco L., Tuffery P., Bomsel M. (2012). Isotype modulates epitope specificity, affinity, and antiviral activities of anti–HIV-1 human broadly neutralizing 2F5 antibody. Proc. Natl. Acad. Sci. USA.

[79] Li T., Tracka M.B., Uddin S., Casas-Finet J., Jacobs D.J., Livesay D.R. (2015). Rigidity emerges during antibody evolution in three distinct antibody systems: Evidence from QSFR analysis of fab fragments. PLoS Comput. Biol..

[80] Sela-Culang I., Alon S., Ofran Y. (2012). A systematic comparison of free and bound antibodies reveals binding-related conformational changes. J. Immunol..

[81] Zhao J., Nussinov R., Ma B. (2017). Allosteric control of antibody-prion recognition through oxidation of a disulfide bond between the CH and CL chains. Protein Eng. Des. Sel..

[82] Lazar G.A., Dang W., Karki S., Vafa O., Peng J.S., Hyun L., Chan C., Chung H.S., Eivazi A., Yoder S.C. (2006). Engineered antibody Fc variants with enhanced effector function. Proc. Natl. Acad. Sci. USA.

[83] Dahiyat B.I., Mayo S.L. (1996). Protein design automation. Protein Sci..

[84] Raha K., Wollacott A.M., Italia M.J., Desjarlais J.R. (2000). Prediction of amino acid sequence from structure. Protein Sci..

[85] Mimura Y., Sondermann P., Ghirlando R., Lund J., Young S.P., Goodall M., Jefferis R. (2001). Role of oligosaccharide residues of IgG 1-Fc in Fc gamma RIIb binding. J. Biol. Chem..

[86] Lu J., Chu J., Zou Z., Hamacher N.B., Rixon M.W., Sun P.D. (2015). Structure of FcgammaRI in complex with Fc reveals the importance of glycan recognition for high-affinity IgG binding. Proc. Natl. Acad. Sci. USA.

[87] Lund J., Tanaka T., Takahashi N., Sarmay G., Arata Y., Jefferis R. (1990). A protein structural change in aglycosylated IgG3 correlates with loss of huFc gamma R1 and huFc gamma R111 binding and/or activation. Mol. Immunol..

[88] Walker M.R., Lund J., Thompson K.M., Jefferis R. (1989). Aglycosylation of human IgG1 and IgG3 monoclonal antibodies can eliminate recognition by human cells expressing Fc gamma Ri and/or Fc gamma RII receptors. Biochem. J..

[89] Jefferis R. (1993). The glycosylation of antibody molecules: Functional significance. Glycoconj. J..

[90] Okbazghi S.Z., More A.S., White D.R., Duan S., Shah I.S., Joshi S.B., Middaugh C.R., Volkin D.B., Tolbert T.J. (2016). Production, characterization, and biological evaluation of well-defined IgG1 Fc glycoforms as a model system for biosimilarity analysis. J. Pharm. Sci..

[91] Subedi G.P., Barb A.W. (2016). The immunoglobulin G1 *N*-glycan composition affects binding to each low affinity Fc gamma receptor. MAbs.

[92] Yamaguchi Y., Nishimura M., Nagano M., Yagi H., Sasakawa H., Uchida K., Shitara K., Kato K. (2006). Glycoform-dependent conformational alteration of the Fc region of human immunoglobulin G1 as revealed by NMR spectroscopy. Biochim. Biophys. Acta.

[93] Shields R.L., Lai J., Keck R., O’Connell L.Y., Hong K., Meng Y.G., Weikert S.H., Presta L.G. (2002). Lack of fucose on human IgG1 n-linked oligosaccharide improves binding to human Fcgamma RIII and antibody-dependent cellular toxicity. J. Biol. Chem..

[94] Lee H.S., Im W. (2017). Effects of n-glycan composition on structure and dynamics of IgG1 Fc and their implications for antibody engineering. Sci. Rep..

[95] Lund J., Takahashi N., Pound J.D., Goodall M., Jefferis R. (1996). Multiple interactions of IgG with its core oligosaccharide can modulate recognition by complement and human Fc gamma receptor I and influence the synthesis of its oligosaccharide chains. J. Immunol..

[96] Yu X., Baruah K., Harvey D.J., Vasiljevic S., Alonzi D.S., Song B.D., Higgins M.K., Bowden T.A., Scanlan C.N., Crispin M. (2013). Engineering hydrophobic protein-carbohydrate interactions to fine-tune monoclonal antibodies. J. Am. Chem. Soc..

[97] Ahmed A.A., Giddens J., Pincetic A., Lomino J.V., Ravetch J.V., Wang L.X., Bjorkman P.J. (2014). Structural characterization of anti-inflammatory immunoglobulin g fc proteins. J. Mol. Biol..

[98] Deisenhofer J. (1981). Crystallographic refinement and atomic models of a human fc fragment and its complex with fragment B of protein a from staphylococcus aureus at 2.9- and 2.8-a resolution. Biochemistry.

[99] Barb A.W., Prestegard J.H. (2011). NMR analysis demonstrates immunoglobulin G *N*-glycans are accessible and dynamic. Nat. Chem. Biol..

[100] Subedi G.P., Barb A.W. (2015). The structural role of antibody *N*-glycosylation in receptor interactions. Structure.

[101] De Groot A.S., Moise L. (2007). New tools, new approaches and new ideas for vaccine development. Expert Rev. Vaccines.

[102] DeLisi C., Berzofsky J.A. (1985). T-cell antigenic sites tend to be amphipathic structures. Proc. Natl. Acad. Sci. USA.

[103] McMurry J., Sbai H., Gennaro M.L., Carter E.J., Martin W., De Groot A.S. (2005). Analyzing mycobacterium tuberculosis proteomes for candidate vaccine epitopes. Tuberculosis.

[104] Sun P., Ju H., Liu Z., Ning Q., Zhang J., Zhao X., Huang Y., Ma Z., Li Y. (2013). Bioinformatics resources and tools for conformational b-cell epitope prediction. Comput. Math. Methods Med..

[105] Sok D., Laserson U., Laserson J., Liu Y., Vigneault F., Julien J.P., Briney B., Ramos A., Saye K.F., Le K. (2013). The effects of somatic hypermutation on neutralization and binding in the PGT121 family of broadly neutralizing HIV antibodies. PLoS Pathog..

[106] Scharf L., Scheid J.F., Lee J.H., West A.P., Chen C., Gao H., Gnanapragasam P.N., Mares R., Seaman M.S., Ward A.B. (2014). Antibody 8ANC195 reveals a site of broad vulnerability on the HIV-1 envelope spike. Cell Rep..

[107] Pejchal R., Doores K.J., Walker L.M., Khayat R., Huang P.S., Wang S.K., Stanfield R.L., Julien J.P., Ramos A., Crispin M. (2011). A potent and broad neutralizing antibody recognizes and penetrates the HIV glycan shield. Science.

[108] Martin W., Sbai H., De Groot A.S. (2003). Bioinformatics tools for identifying class I-restricted epitopes. Methods.

[109] Brusic V., Petrovsky N. (2005). Immunoinformatics and its relevance to understanding human immune disease. Expert Rev. Clin. Immunol..

[110] Korber B., LaBute M., Yusim K. (2006). Immunoinformatics comes of age. PLoS Comput. Biol..

[111] Kringelum J.V., Lundegaard C., Lund O., Nielsen M. (2012). Reliable b cell epitope predictions: Impacts of method development and improved benchmarking. PLoS Comput. Biol..

[112] Sweredoski M.J., Baldi P. (2008). Pepito: Improved discontinuous b-cell epitope prediction using multiple distance thresholds and half sphere exposure. Bioinformatics.

[113] Sun J., Wu D., Xu T., Wang X., Xu X., Tao L., Li Y.X., Cao Z.W. (2009). SEPPA: A computational server for spatial epitope prediction of protein antigens. Nucleic Acids Res..

[114] Burton D.R. (2010). Scaffolding to build a rational vaccine design strategy. Proc. Natl. Acad. Sci. USA.

[115] He L., Zhu J. (2015). Computational tools for epitope vaccine design and evaluation. Curr. Opin. Virol..

[116] Zhou T., Zhu J., Wu X., Moquin S., Zhang B., Acharya P., Georgiev I.S., Altae-Tran H.R., Chuang G.Y., Joyce M.G. (2013). Multidonor analysis reveals structural elements, genetic determinants, and maturation pathway for HIV-1 neutralization by VRC01-class antibodies. Immunity.

[117] Correia B.E., Ban Y.E., Friend D.J., Ellingson K., Xu H., Boni E., Bradley-Hewitt T., Bruhn-Johannsen J.F., Stamatatos L., Strong R.K. (2011). Computational protein design using flexible backbone remodeling and resurfacing: Case studies in structure-based antigen design. J. Mol. Biol..

[118] Correia B.E., Bates J.T., Loomis R.J., Baneyx G., Carrico C., Jardine J.G., Rupert P., Correnti C., Kalyuzhniy O., Vittal V. (2014). Proof of principle for epitope-focused vaccine design. Nature.

[119] Carter P.J. (2006). Potent antibody therapeutics by design. Nat. Rev. Immunol..

[120] Reichert J.M. (2008). Monoclonal antibodies as innovative therapeutics. Curr. Pharm. Biotechnol..

[121] Nelson A.L., Reichert J.M. (2009). Development trends for therapeutic antibody fragments. Nat. Biotechnol..

[122] Fischman S., Ofran Y. (2018). Computational design of antibodies. Curr. Opin. Struct. Biol..

[123] Lensink M.F., Velankar S., Wodak S.J. (2017). Modeling protein-protein and protein-peptide complexes: Capri 6th edition. Proteins.

[124] Clark A.J., Gindin T., Zhang B., Wang L., Abel R., Murret C.S., Xu F., Bao A., Lu N.J., Zhou T. (2017). Free energy perturbation calculation of relative binding free energy between broadly neutralizing antibodies and the GP120 glycoprotein of HIV-1. J. Mol. Biol..

[125] Baker B.M., Murphy K.P. (1998). Prediction of binding energetics from structure using empirical parameterization. Methods Enzymol..

[126] Audie J., Scarlata S. (2007). A novel empirical free energy function that explains and predicts protein-protein binding affinities. Biophys. Chem..

[127] Camacho C.J., Vajda S. (2001). Protein docking along smooth association pathways. Proc. Natl. Acad. Sci. USA.

[128] Dell’Orco D., De Benedetti P.G., Fanelli F. (2007). In silico screening of mutational effects on enzyme-proteic inhibitor affinity: A docking-based approach. BMC Struct. Biol..

[129] Zhang C., Liu S., Zhu Q., Zhou Y. (2005). A knowledge-based energy function for protein-ligand, protein-protein, and protein-DNA complexes. J. Med. Chem..

[130] Sirin S., Apgar J.R., Bennett E.M., Keating A.E. (2016). Ab-bind: Antibody binding mutational database for computational affinity predictions. Protein Sci..

[131] Piekarska B., Drozd A., Konieczny L., Krol M., Jurkowski W., Roterman I., Spolnik P., Stopa B., Rybarska J. (2006). The indirect generation of long-distance structural changes in antibodies upon their binding to antigen. Chem. Biol. Drug Des..

[132] Bowen A., Casadevall A. (2016). Revisiting the immunoglobulin intramolecular signaling hypothesis. Trends Immunol..

[133] Oda M., Kozono H., Morii H., Azuma T. (2003). Evidence of allosteric conformational changes in the antibody constant region upon antigen binding. Int. Immunol..

[134] Lane T.J., Bowman G.R., Beauchamp K., Voelz V.A., Pande V.S. (2011). Markov state model reveals folding and functional dynamics in ultra-long md trajectories. J. Am. Chem. Soc..

[135] Husic B.E., Pande V.S. (2018). Markov state models: From an art to a science. J. Am. Chem. Soc..

